# Appointments and Changes in Medical Schools

**Published:** 1853-06

**Authors:** 


### APPOINTMENTS AND CHANGES IN MEDICAL SCHOOLS.

The chair of Anatomy in the University of Pennsylvania, vacated by the death of Prof. Horner, has been filled by the appointment of Dr. Joseph Leidy, who completed the anatomical course in that school, last winter, after the failure of Prof. H.'s health. Dr. Leidy has no superior in our country as a scientific anatomist. He has evinced a strong taste for comparative anatomy, and his labors in that department have already conferred upon him a high reputation. We rejoice in the promotion of a young man who, as in this instance, is wholly indebted for his success to his talents, and unflinching industry.

Dr Clymer has resigned the chair of Theory and Practice of Medicine in the University of New York, and is succeeded by Dr. Swett, of that City. This appointment cannot fail to give satisfaction to the profession of the metropolis, and will be hailed by its members abroad as a measure calculated to strengthen the school. Dr. Swett is one of the "solid" men of the medical, profession, skilful and learned in practical medicine, whose teachings will be received by his pupils as the result of matured experience, and patient, discriminating observation at the bed side.

Dr Willis G. Edwards has been compelled by declining health to resign the chair of Clinical Medicine and Pathological Anatomy in the University of St. Louis. Dr John B. Johnston, who, for a number of years, has been connected with the University of Missouri, is his successor. Dr Gratz. Dr. Moses, and Dr Barnett, have also resigned their places in the University of Missouri, and are succeeded by Dr Barnes. Dr Kavanaugh, and Dr Deming. The chairs of Anatomy and physiology, heretofore connected in that school with other chairs, have been erected into distinct professorships, and Dr John Hodgen, and Dr Payton Spencer elected to them.

Prof. Rogers, and Prof. J. Lawrence Smith have resigned their chairs in the University of Virginia.

At the time that Prof. Austin Flint was elected to the chair of Theory and Practice of Medicine in the University of Louisville, he held the corresponding chair in the University of Buffalo, and during the last winter and spring delivered a full course of lectures in both institutions. He has recently resigned his professorship in the latter. Prof. F. will unite with his colleagues in the October course of lectures in the University of Louisville.

